# Suppression of adaptive immunity to heterologous antigens during *Plasmodium *infection through hemozoin-induced failure of dendritic cell function

**DOI:** 10.1186/jbiol34

**Published:** 2006-04-12

**Authors:** Owain R Millington, Caterina Di Lorenzo, R Stephen Phillips, Paul Garside, James M Brewer

**Affiliations:** 1 Division of Immunology, Infection and Inflammation, University of Glasgow, Glasgow G11 6NT, UK; 2 Division of Infection and Immunity, Joseph Black Building, University of Glasgow, Glasgow G12 8QQ, UK; 3 Current address: Centre for Biophotonics, University of Strathclyde, Glasgow G4 0NR, UK

## Abstract

**Background:**

Dendritic cells (DCs) are central to the initiation and regulation of the adaptive immune response during infection. Modulation of DC function may therefore allow evasion of the immune system by pathogens. Significant depression of the host's systemic immune response to both concurrent infections and heterologous vaccines has been observed during malaria infection, but the mechanisms underlying this immune hyporesponsiveness are controversial.

**Results:**

Here, we demonstrate that the blood stages of malaria infection induce a failure of DC function *in vitro *and *in vivo*, causing suboptimal activation of T cells involved in heterologous immune responses. This effect on T-cell activation can be transferred to uninfected recipients by DCs isolated from infected mice. Significantly, T cells activated by these DCs subsequently lack effector function, as demonstrated by a failure to migrate to lymphoid-organ follicles, resulting in an absence of B-cell responses to heterologous antigens. Fractionation studies show that hemozoin, rather than infected erythrocyte (red blood cell) membranes, reproduces the effect of intact infected red blood cells on DCs. Furthermore, hemozoin-containing DCs could be identified in T-cell areas of the spleen *in vivo*.

**Conclusion:**

*Plasmodium *infection inhibits the induction of adaptive immunity to heterologous antigens by modulating DC function, providing a potential explanation for epidemiological studies linking endemic malaria with secondary infections and reduced vaccine efficacy.

## Background

Malaria is the major parasitic disease of humans throughout the tropics and subtropics, mainly affecting children under 5 years of age and causing 500 million clinical cases and up to 2.7 million deaths each year [1]. In addition to infection-induced mortality, malaria is also associated with public-health problems resulting from impairment of immune responses. Although this immunosuppression may have evolved as a mechanism by which the parasite can prevent immune-mediated clearance [2-8], it leaves malaria-infected individuals or experimental animals more susceptible to secondary infections, such as non-typhoidal *Salmonella *[9], herpes zoster virus [10], hepatitis B virus [11], Moloney leukemia virus [12] and nematode infection [13], as well as Epstein-Barr virus reactivation [14-17]. Because the efficacy of heterologous vaccines can also be suppressed in malaria-infected patients [18-21], children showing clinical signs of malaria are rarely immunized until after anti-malarial chemoprophylaxis, which can improve the response to vaccination [22]. In a recent study of a new conjugate vaccine against pneumococci, efficacy was reduced during the malaria transmission season [23], demonstrating the possible impact of malaria infection on large-scale vaccine regimes. Certain vaccines, however, seem to induce protective responses irrespective of malaria status and the immunosuppressive effect of malaria infection might thus not extend to all antigens [20]; studies *in vivo *are required to investigate this controversy further. Several animal studies have described suppression of immune function by *Plasmodium *parasites *in vitro *and *in vivo *[24-34], but the mechanisms involved remain unclear.

Dendritic cells (DCs) have a crucial role in the activation of T cells and consequently in the induction of adaptive immune responses and immunity [35,36]. There is evidence that many pathogens have evolved mechanisms that subvert DC function, thereby modulating the host's immune response to their advantage [37,38]. Recent studies have revealed that DCs are important in malaria infection, particularly during the early events of induction of the protective immune response to infection [39,40]. It has been reported that red blood cells (RBCs) infected with schizont-stage *Plasmodium falciparum *activate plasmacytoid DCs as detected by increased expression of the antigen CD86 and the cytokine interferon-α (IFN-α) *in vitro *[41]. In contrast, the asexual erythrocytic stages of *P. falciparum *were shown to impair the ability of human DCs to undergo maturation *in vitro *[42]. Indeed, peripheral blood DCs of *P. falciparum*-infected children showed reduced levels of the major histocompatibility complex (MHC) molecule HLA-DR compared with uninfected controls [43], suggesting a reduced activation state. Thus, the ability of malaria parasites to inhibit maturation of DCs could be involved not only in parasite-specific immunosuppression but also in the suppression of responses to heterologous antigens such as vaccines and unrelated pathogens [2,19,20]. As human malaria parasites are host-specific, however, observations on the effect of human malaria on DCs are largely limited to studies *in vitro*.

Here, we describe the mechanism underlying this suppression of immunity *in vitro *and *in vivo*. DC activation is dynamically altered by parasitized erythrocytes (pRBCs), partly because of deposition of the malarial pigment hemozoin (HZ) within these cells. Following presentation of heterologous antigen by pRBC-exposed DCs, there is less expansion of CD4^+ ^'helper' T cells that are essential for the induction of adaptive immunity. Subsequently, migration of T cells to lymphoid follicles is abrogated, leading to defective B-cell expansion and differentiation and a failure of the antibody response. These studies explain why immunity to malaria is slow to develop and why protection against secondary infections is reduced in *Plasmodium*-infected individuals.

## Results

### Suppression of heterologous immune responses during malaria infection

**Figure 1:**
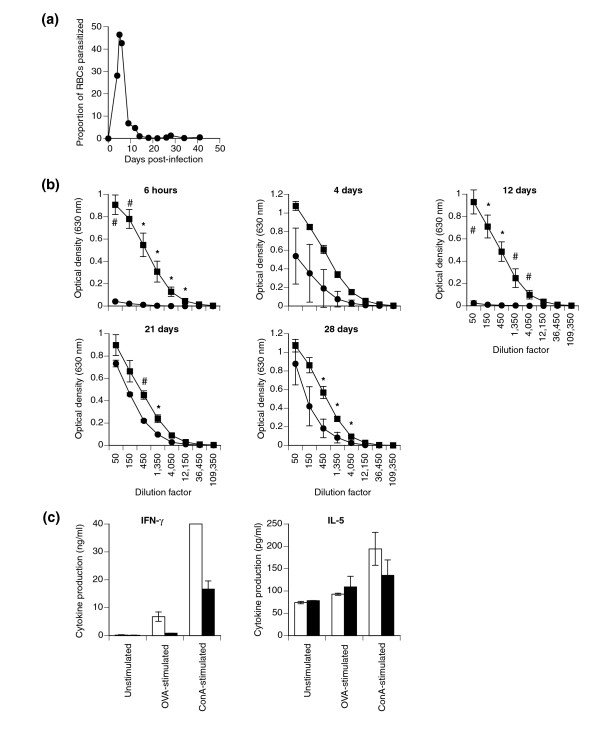
Suppression of immunity by *P. chabaudi *infection. **(a) **BALB/c mice were infected with 10^6 ^*P. chabaudi *(AS strain) parasites by intra-peritoneal injection and the proportion of peripheral blood cells parasitized (parasitemia) was monitored by Giemsa's stain of peripheral blood smears. **(b) **Uninfected (squares) or *P. chabaudi*-infected (circles) BALB/c mice were immunized with OVA/LPS at the indicated times after infection. Three weeks after immunization, sera were analyzed for OVA-specific IgG. Data represent the mean of three mice per group ±1 standard deviation (s.d.) and are representative of two similar experiments (*p ≤ 0.05, ^#^*p *≤ 0.005 uninfected versus *P. chabaudi*-infected). **(c) **Spleen cells from uninfected (open bars) or *P. chabaudi*-infected (filled bars) BALB/c mice immunized with OVA/LPS 10 days post-infection were restimulated *in vitro *as indicated and supernatants assayed for levels of IFN-γ (left) and IL-5 (right) after 48 h. Uninfected, immunized animals generated OVA-specific IgG responses, but OVA and LPS administered 6 hours and 12 days after infection with *P. chabaudi *produced significantly reduced levels of IgG (Figure 1b). Interestingly, suppression was lower in mice immunized during increasing levels of parasite infection (parasitemia; Figure 1b, 4 days). By 21 days post-infection, infected animals had regained immune responsiveness and mounted antibody responses of a similar magnitude to those seen in uninfected controls (Figure 1b, 21 and 28 days). Production of OVA-stimulated and concanavalin A (ConA) mitogen-stimulated T-cell cytokines was reduced in cultures of splenocytes taken from mice infected with *P. chabaudi *10 days before immunization (Figure 1c). Thus, as described for *P. falciparum *in humans, *P. chabaudi *infection in mice induces suppression of immune responses, although these studies reveal that, in the animal model at least, this is a highly dynamic phenomenon

We first examined the response to a heterologous antigen during *Plasmodium chabaudi *(AS strain) infection (Figure 1a) to determine whether this murine model reflected the clinical immunosuppression observed with *P. falciparum *infection [18-21]. Mice were immunized with the model antigen ovalbumin (OVA) and lipopolysaccharide (LPS) to act as adjuvant at various times after infection, and OVA-specific serum immunoglobulin G (IgG) was measured 21 days later.

### Modulation of DCs *in vitro *by infected erythrocytes

DC activation is central to induction of adaptive immunity [35], and previous studies have suggested that several protozoan pathogens have evolved mechanisms to suppress this response and consequently to reduce immune-mediated protection [44]. Human DCs cultured with *P. falciparum*-infected erythrocytes are hyporesponsive to stimulation with LPS and less capable of stimulating CD4^+ ^T-cell responses [42]. This observation remains controversial, however, as studies using murine models have suggested that DCs may be activated during increasing parasitemia *in vivo *[45] and following culture *in vitro *with parasite schizont-infected erythrocytes [40].

**Figure 2:**
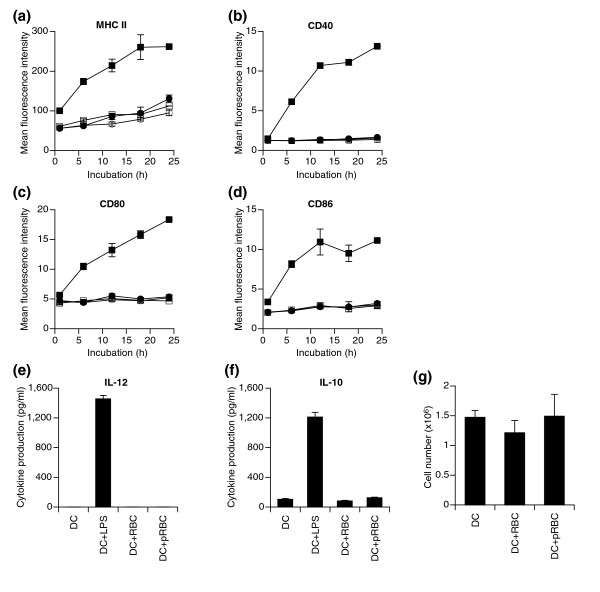
*P. chabaudi*-infected erythrocytes do not activate DCs directly *in vitro*. DCs (2 × 10^6^) were cultured with 2 × 10^8 ^infected erythrocytes (DC+pRBC; filled circles) or with an equal number of uninfected erythrocytes (DC+RBC; empty circles). Control DCs remained unstimulated (DC; empty squares) or were stimulated with 1 μg/ml of LPS (DC+LPS; filled squares). Results show the mean fluorescence intensity of **(a) **MHC class II, **(b) **CD40, **(c) **CD80, and **(d) **CD86, as determined by FACS analysis of gated CD11c^+ ^cells at various times of co-culture. Supernatants were analyzed for concentrations of **(e) **IL-12 (p40 subunit) and **(f) **IL-10 secreted by DCs after 24 h of incubation. **(g) **The number of viable DCs was determined by trypan blue exclusion. Results show the mean value ± standard error (s.e.) of triplicate samples per group.

As the results above indicated that immune responsiveness *in vivo *is dynamically regulated during infection, we studied the ability of *P. chabaudi*-infected erythrocytes to modulate DCs directly, by examining the expression of MHC class II and co-stimulatory molecules on DCs. Bone-marrow-derived DCs were incubated with infected erythrocytes and the expression of surface markers examined at various times over the following 24 hours. Our results show that DCs expressed very low levels of surface MHC class II and the co-stimulatory molecules CD40, CD80 and CD86 when cultured in growth medium alone, thus confirming the immature state of these DCs in culture (Figure 2a-d). Stimulation with LPS promoted a significant increase in the expression level of all co-stimulatory molecules within 6 hours. DCs incubated with RBCs or pRBCs did not, however, increase expression of MHC class II, CD40, CD80 and CD86, indicating that malaria parasites do not induce DC activation directly. Analysis of cytokine production showed that DCs exposed to RBC or pRBCs produce small, though detectable, amounts of both interleukins IL-12 and IL-10 that are a thousand-fold lower than those observed after LPS stimulation (Figure 2e,f), suggesting that the presence of parasites does not result in DC activation. The viability of treated DCs and control cells was quantified after 24 hours of culture by trypan blue exclusion (Figure 2g) and propidium iodide (PI) and annexin V staining (data not shown) and was not significantly affected by pRBCs.

**Figure 3:**
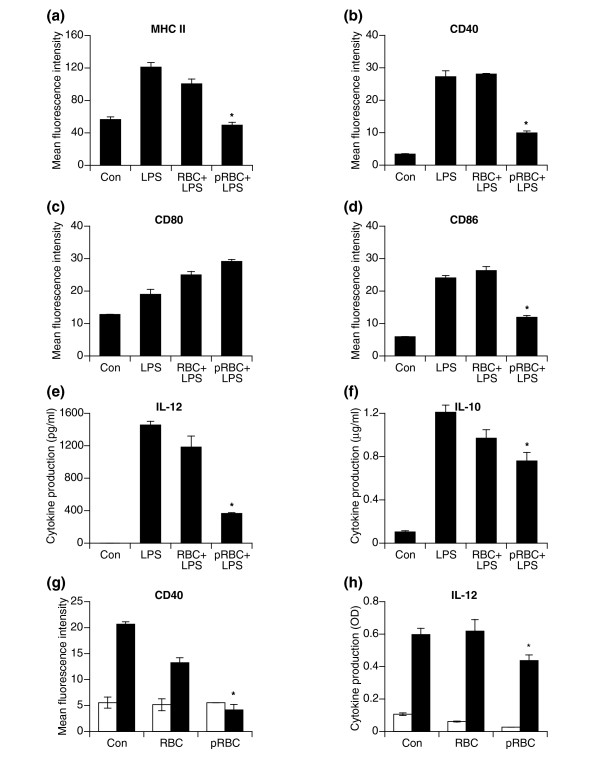
*P. chabaudi*-infected erythrocytes inhibit the LPS and CD40L-induced maturation of DCs. DCs were cultured with infected (pRBC) or uninfected (RBC) erythrocytes for 24 h, as described in Figure 2, then treated with 1 μg/ml of LPS for a further 18 h. **(a-f) **Results were analyzed as described in Figure 2 (*p ≤ 0.05 pRBC versus RBC). **(g,h) **DCs (1 × 10^6^) were cultured with 1 × 10^8 ^infected erythrocytes (pRBC) before stimulation with CD40L-expressing fibroblasts (filled bars) or control fibroblasts (open bars) at a 1:1 ratio of fibroblasts:DCs. Untreated DCs (Con) and uninfected erythrocyte-treated DCs (RBC) were used as controls. DCs were incubated with fibroblasts for a further 18 h before analyzing CD40 expression and cytokine production. CD40 expression on DCs is shown as the mean fluorescent intensity on CD11c^+ ^cells. IL-12 production is shown as the mean optical density (OD) read at 450 nm. Results show the mean value ± s.e. of triplicate samples per group (*p ≤ 0.05 pRBC versus RBC).

Having established that pRBCs at a ratio of 100:1 do not directly induce DC maturation, we examined whether pRBC-treated DCs retained their ability to mature in response to LPS treatment *in vitro*. DCs were exposed to RBCs or pRBCs for 24 hours and subsequently challenged with LPS. After 18 hours of LPS stimulation, the expression levels of MHC class II, CD40, CD80 and CD86 increased significantly (Figure 3a-d). DCs pre-incubated with pRBCs and subsequently challenged with LPS, however, showed significantly lower levels of expression of MHC class II, CD40, and CD86 compared with those observed when cells were not treated with pRBCs or were pre-incubated with RBCs before the LPS challenge (Figure 3a-d). Kinetic studies demonstrated that the ability of pRBCs to induce this hyporesponsive state in DCs required at least 6 hours pre-incubation before the addition of LPS (data not shown). Cytokine production following treatment with LPS showed that, although DCs treated with pRBCs could still produce appreciable levels of IL-12 and IL-10 in response to LPS, the amount produced was significantly lower than that produced by DCs pre-incubated with RBCs (Figure 3e,f). As the interaction between CD40 on DCs and its ligand CD40L on T cells *in vivo *is known to be crucial in the production of bioactive IL-12 and upregulation of adhesion and co-stimulatory molecules [46,47], we stimulated bone-marrow-derived DCs with CD40L-transfected fibroblasts (Figure 3g,h). DCs treated with RBCs significantly upregulated CD40 expression in response to CD40L and produced high levels of the inducible IL-12p40 subunit. CD40 ligation, however, did not rescue the reduced maturation of DCs treated with pRBCs, although, as previously observed with the LPS treatment, these cells still produced IL-12 p40 but to a lesser extent than the control groups.

### Modulation of DCs *in vivo *during malaria infection

**Figure 4:**
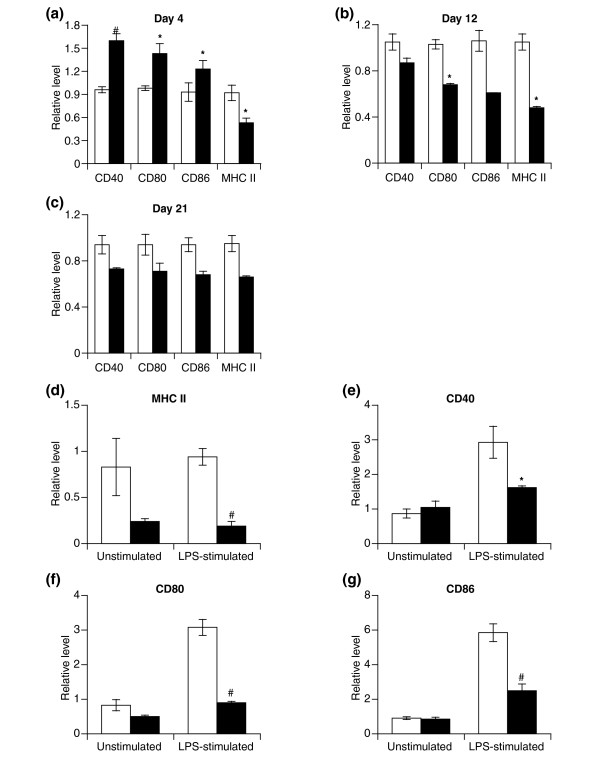
Modulation of DC activation *in vivo *during *P. chabaudi *infection. **(a-c) **Splenic CD11c^+ ^DCs were isolated from *P. chabaudi *infected mice at various times (filled bars) or from uninfected controls (open bars) and analyzed by flow cytometry for the indicated markers. Data are expressed as mean fluorescence intensity relative to uninfected samples ± 1 s.d. (*p ≤ 0.05 uninfected versus *P. chabaudi*-infected). **(d-g) **Splenic DCs from uninfected (open bars) or *P. chabaudi*-infected (filled bars; 12 days post-infection) BALB/c mice were restimulated *in vitro *with LPS (1 μg/ml) for 18 h before analysis by flow cytometry for (d) MHC class II, (e) CD40, (f) CD80 and (g) CD86. Data are expressed as mean fluorescence intensity relative to uninfected, unstimulated samples ± 1 s.d. (*p ≤ 0.05, ^#^*p *≤ 0.005 uninfected versus *P. chabaudi*-infected for the same treatment).

As our results suggested that malaria-infected erythrocytes might modulate the responsiveness of DCs *in vitro*, we next investigated the activation status of splenic DCs *in vivo *during a time-course of infection with *P. chabaudi*. DCs isolated from spleens of mice 4 days after infection showed a moderately activated phenotype, as demonstrated by increased expression of CD40 and CD80 (Figure 4a), confirming previous reports [45]. DCs isolated from infected animals 12 and 20 days after infection, however, showed a reduced level of activation, with lower levels of CD40, CD80, CD86 and MHC class II molecules on their surface compared with DCs from uninfected animals (Figure 4b,c). Whereas DCs from uninfected mice upregulated CD40, CD80 and CD86 following LPS stimulation (Figure 4d-g), DCs isolated from the spleens of *P. chabaudi*-infected mice remained refractory to *in vitro *LPS-induced maturation, with reduced levels of these molecules following stimulation. Thus it seems that, *in vivo*, DCs are activated soon after infection, and the level of activation on DCs is reduced following the peak of infection (days 12-20), and this cannot be abrogated by microbial stimulation *ex vivo*.

### Identification of *P. chabaudi *components that induce DC hyporesponsiveness

**Figure 5:**
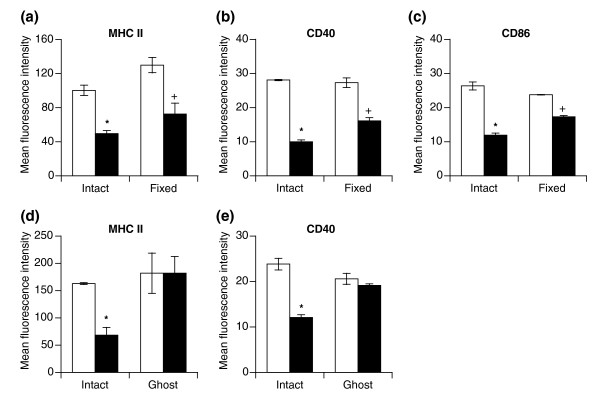
Identification of *P. chabaudi *components that inhibit LPS-induced DC maturation. **(a-c) **DCs (2 × 10^6^) were treated for 24 h with uninfected RBC (open bars) or infected RBC (pRBC; filled bars) that were untreated or fixed before stimulation with LPS (1 μg/ml) for 18 h. DC activation was characterized by analysis of (a) MHC class II, (b) CD40, and (c) CD86 expression on CD11c^+ ^DC surfaces by FACS (*p ≤ 0.05 pRBC versus RBC, +*p *≤ 0.05 fixed pRBC versus fixed RBC). **(d,e) **DCs (5 × 10^5^) were incubated with intact erythrocytes or RBC ghosts from uninfected (open bars) or infected erythrocytes (filled bars), for 24 h before stimulation with LPS for 18 h. DC activation was characterized by analysis of (d) MHC class II and (e) CD40 expression on the DC surface by FACS (*p ≤ 0.05 pRBC versus RBC).

As we had demonstrated that malaria infection modulates key aspects of DC function, we wanted to examine the possible mechanisms involved. Initially, to determine whether maturation of the parasite from the trophozoite stage to the schizont stage *in vitro *or some metabolic process within the pRBCs was required for induction of DC hyporesponsiveness, pRBCs were fixed with paraformaldehyde and incubated with DCs for 24 hours before the addition of LPS (Figure 5a-c). The expression levels of MHC class II, CD40 and CD86 were significantly reduced when DCs were co-cultured with fixed pRBCs before LPS challenge, confirming that trophozoite-infected erythrocytes downregulate DC activation in response to LPS treatment and suggesting that growth and maturation of the parasite into schizonts is not required for suppression. In an attempt to understand the mechanisms involved in the parasite-mediated modulation of DC function, we investigated the role of selected parasite components on the LPS-induced maturation of DCs. We addressed this question initially by analyzing the effect that parasite proteins expressed on the erythrocyte surface membrane have on DC function. DCs were exposed to RBC membranes (ghosts) isolated from infected or uninfected erythrocytes. After 24 hours of culture, DCs were challenged with LPS and the expression of co-stimulatory molecules analyzed 18 hours later (Figure 5d,e). Our results clearly show that ghosts isolated from pRBCs did not alter the ability of DCs to respond to LPS treatment *in vitro*, as observed by the levels of MHC class II and CD40, demonstrating that proteins expressed on the surface membranes of pRBCs are apparently not essential for the modulation of DC function.

**Figure 6:**
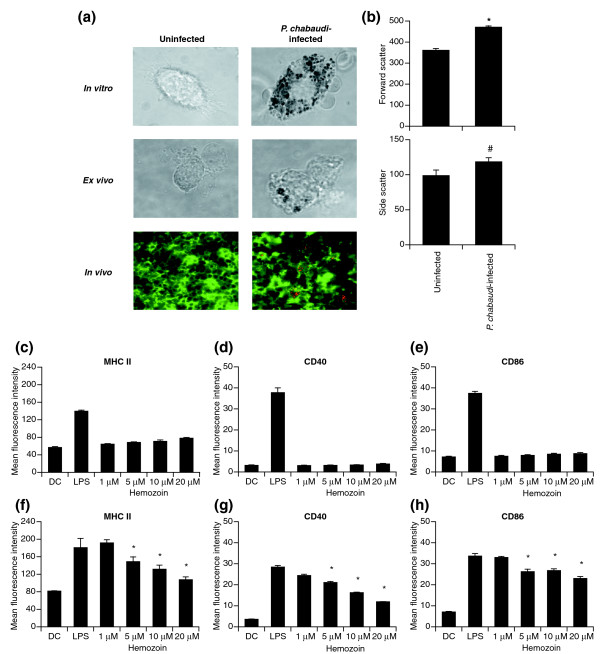
Deposition of HZ in DCs suppresses maturation. **(a) **HZ content in bone-marrow-derived DCs (*In vitro*), purified DCs (*Ex vivo*) and spleen sections (*In vivo*) was visualized by light microscopy (top images) or by false-coloring malaria pigment viewed in bright-field image (red) and superimposing over the fluorescent CD11c image (green). **(b) **CD11c^+ ^DCs were analyzed for size and granularity by flow cytometry 12 days post-infection with *P. chabaudi *or in uninfected controls. Data are expressed as the mean forward scatter or side scatter of triplicate samples ± 1 s.d. (*p ≤ 0.05, ^#^*p *≤ 0.005 uninfected versus *P. chabaudi*-infected). **(c-e) **2 × 10^6 ^DCs were cultured with 1 μM, 5 μM, 10 μM and 20 μM of HZ. After 24 h, the level of expression of (c) MHC class II, (d) CD40 and (e) CD86 on CD11c^+ ^cells was determined by FACS analysis. **(f-h) **After 24 h culture with HZ, 1 μg/ml LPS was added to DCs and the levels of (f) MHC class II, (g) CD40, and (h) CD86 were analyzed 18 h later by FACS. All results are shown as the mean fluorescence intensity on CD11c^+ ^DCs in triplicate samples ± s.e. (^*^p ≤ 0.05 HZ versus LPS).

Having established that parasite proteins expressed on the erythrocyte cell membrane are not responsible for the modulation of LPS-induced maturation of DCs *in vitro*, we focused our attention on HZ, a by-product of hemoglobin digestion. We observed that bone-marrow-derived DCs cultured *in vitro *with infected erythrocytes accumulated intracellular malarial pigment (Figure 6a). Flow cytometric analysis of splenic DCs, identified by CD11c expression, also demonstrated an increase in the size and granularity of DCs during infection (Figure 6b), and DCs isolated *ex vivo *as well as DCs in spleen sections showed conspicuous HZ deposition (Figure 6a).

In order to assess the role of HZ in the parasite-induced modulation of DC function, we initially analyzed its ability to activate DCs directly *in vitro *(Figure 6c-e). Even at the highest dose (20 μM), HZ did not induce DC maturation, as the levels of MHC class II, CD40 and CD86 were the same as the levels expressed by untreated controls. We then examined whether HZ-treated DCs still responded to LPS treatment *in vitro *(Figure 6f-h). Our data clearly demonstrate a dose-dependent inhibition of the LPS-induced maturation of DCs by HZ, as seen by the reduced levels of MHC class II, CD40 and CD86. Taken together, these results indicate that HZ, rather than pRBC membranes, is a key factor involved in the suppression of murine DC function *in vitro *and *in vivo*.

### Failure of pRBC-treated DCs to induce T-cell effector function

**Figure 7:**
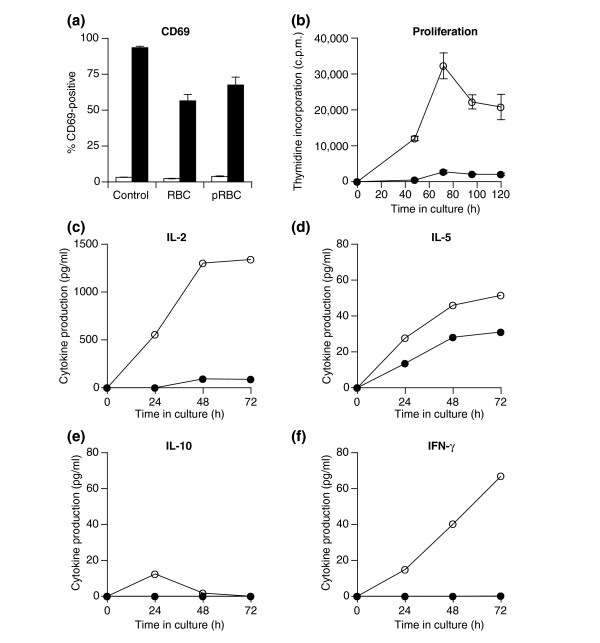
*P. chabaudi*-infected erythrocytes inhibit the ability of DCs to efficiently activate naive T cells *in vitro*. DCs (2 × 10^5^) were treated with infected (pRBC) or uninfected (RBC) erythrocytes for 24 h. DCs were then loaded with 5 mg/ml of OVA for 6 h. 5 × 10^5 ^untreated (Control), RBC or pRBC-treated DCs and 5 × 10^5 ^DO11.10 lymph node cells were then co-cultured. **(a) **CD69 expression assessed on DO11.10 T cells 24 h later by FACS analysis. Results are expressed as the percentage of antigen-specific cells expressing CD69 in cultures stimulated by OVA-pulsed DCs (filled bars) or by DCs only (open bars). Results show the mean of triplicate samples ± s.e. **(b) **[^3^H]thymidine was added for the last 18 h of culture; c.p.m., counts per min. Results show mean proliferation of OVA-specific T cells after incubation with DCs treated with uninfected RBCs (open circles) or pRBCs (filled circles) in triplicate samples ± s.e. The concentrations of **(c) **IL-2, **(d) **IL-5 **(e) **IL-10, and **(f) **IFN-γ secreted by OVA-specific T cells after incubation with DCs treated with uninfected RBCs (open circles) or pRBCs (filled circles) were measured in supernatants harvested after 24, 48, and 72 h of culture.

The above experiments clearly implicated HZ-mediated suppression of DC function in the failure of antibody production and in the delayed acquisition of protective immunity seen during malaria infection *in vivo*. To examine the functional consequences of malaria on DC function directly, we examined the ability of affected DCs to activate naive, OVA-specific T-cell receptor-transgenic T cells. These cells allow monitoring of the antigen-specific CD4^+ ^T-cell response as their antigen specificity is known and they can be tracked using a clonotypic antibody directed against their T-cell receptor. One of the earliest cell-surface antigens expressed by T cells following activation is CD69, which is detectable within an hour of ligation of the T-cell receptor complex [48]. Interestingly, there was no significant difference in the percentage of OVA-specific T cells expressing CD69 between RBC-treated and pRBC-treated groups (Figure 7a), showing that T cells interacting with modulated DCs are equally activated.

To investigate whether treatment of DCs with pRBCs could alter the dynamics of the T-cell proliferative response, we harvested the T cells at 48, 72, 96 and 120 hours of culture (Figure 7b). The ability of pRBC-treated DCs to induce T-cell proliferation was dramatically reduced compared to the control group throughout the observation period (see Figure 7b). Analysis of T-cell production of IL-2, IL-5, IL-10 and IFN-γ (Figure 7c-f) revealed that each cytokine was downregulated in the pRBC-treated groups compared with RBC-treated controls. The observed reduction in T-cell proliferation and cytokine production could not be explained by T-cell death, as similar levels of necrotic and apoptotic T cells were detected in both conditions by FACS analysis of propidium iodide and annexin staining (data not shown). Thus, although DCs pre-treated with pRBCs *in vitro *can induce initial activation of naive CD4^+ ^T cells, causing upregulation of CD69, these T cells fail to proliferate effectively and have a reduced ability to secrete effector cytokines.

### Suppression of T- and B-cell proliferation during malaria infection

**Figure 8:**
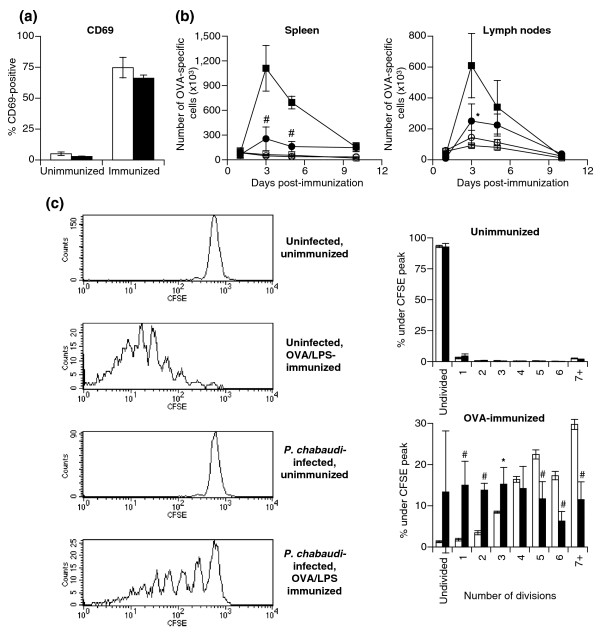
Suppression of CD4^+ ^T-cell expansion by *P. chabaudi *infection *in vivo*. **(a) **Uninfected (open bars) or *P. chabaudi*-infected (filled bars) BALB/c mice received DO11.10 T cells and remained unimmunized or were immunized with OVA/LPS 12 days post-infection. Expression of CD69 on splenic OVA-specific T cells was assessed 18 h post-immunization. Results shown are the proportion of OVA-specific CD4^+^KJ1.26^+ ^T cells expressing CD69 and represent the mean of three mice per group ±1 s.d. **(b) **Uninfected (squares) or *P. chabaudi*-infected (circles) BALB/c mice were transferred and immunized (filled symbols) or remained unimmunized (open symbols) and the absolute number of OVA-specific T cells in spleen (left) and lymph nodes (right) calculated at various times. Results show the mean number of CD4^+^KJ1.26^+ ^T cells and represent the mean of three mice per group ±1 s.d. (*p ≤ 0.05, ^#^*p *≤ 0.005 uninfected and immunized versus *P. chabaudi*-infected and immunized). **(c) **Representative CFSE profiles of OVA-specific T cells are shown (left) and data shown as the mean proportion of CD4^+^KJ1.26^+ ^cells under each CFSE peak in uninfected (open bars) and *P. chabaudi*-infected (filled bars) following immunization (bottom histogram) or in unimmunized controls (upper histogram). Data represent the mean of 3 mice per group ±1 s.d. (*p ≤ 0.05, ^#^*p *≤ 0.005 uninfected and immunized versus *P. chabaudi*-infected and immunized).

To characterize fully the downstream effects of HZ-induced DC hyporesponsiveness on heterologous immune responsiveness, we directly investigated antigen-specific T-cell responses *in vivo *by transferring traceable OVA-specific CD4^+ ^T cells into recipient mice [49]. This allows us to follow the response of a small, but detectable, number of antigen-specific CD4^+ ^T cells during the induction of an adaptive immune response. Cells were transferred into recipients following the initial peak of parasitemia (day 12 of infection) and immunized 1 day later. In *P. chabaudi*-infected immunized mice, OVA-specific CD4^+ ^T cells underwent a similar initial activation to that of antigen-specific T cells in uninfected mice, as estimated by upregulation of CD69 (Figure 8a) and increased size/blastogenesis (also an indicator of activation; data not shown), confirming the *in vitro *observation (see Figure 7a). Antigen-specific CD4^+ ^T cells in lymph nodes and in spleen failed to expand to the same extent in infected mice as in uninfected controls (Figure 8b), however, partly because the antigen-specific CD4^+ ^T cells made a reduced number of divisions (Figure 8c). Interestingly, we did not detect increased apoptosis (determined by annexin staining) of OVA-specific T cells transferred into malaria-infected individuals (data not shown).

**Figure 9:**
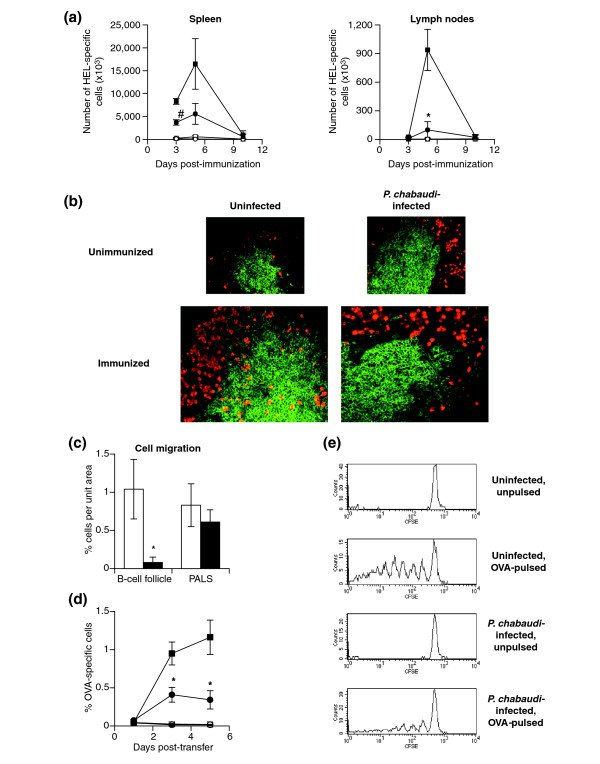
*P. chabaudi *infection causes reduced expansion of B cells and failure of CD4^+ ^T-cell migration through modulation of DCs. **(a) **Uninfected (squares) or *P. chabaudi*-infected (circles) BALB/c mice received OVA-specific CD4^+ ^T cells and HEL-specific B cells 12 days after infection and were immunized with OVA-HEL/LPS (filled symbols) 24 h later. Controls remained unimmunized (open symbols). Results shown are the absolute number of HEL-specific B cells in the spleen (left) and lymph nodes (LN, right) and represent the mean of 3 mice per group ±1 s.d. and are representative of 3 similar experiments (*p ≤ 0.05, ^#^*p *≤ 0.005 uninfected and immunized versus *P. chabaudi*-infected immunized). **(b) **Five days after immunization, spleens from the mice described in (a) were snap frozen and prepared for immunohistochemistry. Sections were stained using biotinylated-KJ1.26 followed by Streptavidin-AlexaFluor 647 to detect OVA-specific T cells (red) and B220-FITC to identify B-cell areas (green). Images shown are representative of 3 mice per group from 2 similar experiments. **(c) **Sections stained as in (b) were analyzed by laser-scanning cytometry. Results are expressed as mean proportion of OVA-specific T cells per unit area in the regions indicated. The number of OVA-specific T cells contained in identically sized regions of follicle and periarteriolar lymphoid sheath (PALS) was calculated and expressed as a proportion of total KJ1.26^+ ^cells in the section to avoid bias due to the difference in expansion between uninfected and infected spleens. Results represent triplicate readings of 3 mice per group ±1 s.d. (*p ≤ 0.05 uninfected and immunized versus *P. chabaudi*-infected and immunized). **(d) **Bone-marrow-derived DCs were cultured with *P. chabaudi *pRBCs (circles) or RBCs (squares) for 18 h before pulsing with 5 mg/ml OVA (filled symbols). Controls remained unpulsed (open symbols). OVA-specific CD4^+ ^T cells were then added at a ratio of 1:1 and cultured for 72 h *in vitro*. T cells were isolated, washed and transferred into uninfected recipients immunized 48 h before transfer to synchronize the immune response. Clonal expansion was then assessed as described above. Results show the mean proportion of CD4^+^KJ1.26^+ ^T cells and represent the mean of 3 mice per group ±1 s.d. (*p ≤ 0.05, RBC-cultured, OVA-pulsed DCs versus pRBC-cultured, OVA-pulsed DC). **(e) **DCs were purified from spleens of uninfected or *P. chabaudi*-infected mice and pulsed with 5 mg/ml OVA for 2 h. Cells were then harvested, washed and 5 × 10^5 ^DCs transferred into uninfected BALB/c mice along with CFSE-labeled OVA-specific T cells. The level of CFSE in OVA-specific CD4^+ ^cells was analyzed 5 days later, as described in Figure 8.

One of the most significant components of CD4^+ ^T-cell effector function is migration into primary lymphoid follicles to interact with, and provide help for, antigen-specific B cells [50]. To track the effect of malaria infection on these populations, we transferred B-cell-receptor transgenic B cells specific for hen egg-white lysozyme (HEL) taken from the MD4 transgenic mouse, together with the OVA-specific DO11.10 T cells and immunized with OVA coupled to HEL [51]. B-cell expansion in uninfected animals peaked 5 days after immunization (Figure 9a). Expansion of HEL-specific B cells was almost completely ablated in *P. chabaudi*-infected animals immunized with OVA-HEL/LPS (Figure 9a), however, suggesting a defect in B-cell activation and/or T-cell help in infected mice.

### Failure of heterologous antigen-specific T-cell migration during malaria infection

Optimal expansion of antigen-specific B cells requires their cognate interaction with CD4^+ ^T cells [52,53]; we therefore examined the localization of OVA-specific CD4^+ ^T cells following immunization. Five days after immunization of uninfected mice, clonal expansion of antigen-specific T cells was evident by immunohistochemistry, and these cells had begun to migrate into B-cell follicles (Figure 9b). In *P. chabaudi*-infected mice, however, not only were there reduced numbers of OVA-specific T cells, but these cells were almost completely excluded from B-cell follicles (see Figure 9b). Migration was quantified using laser-scanning cytometry [54,55]. As shown in Figure 9c, the average proportion of OVA-specific CD4^+ ^T cells in B-cell follicles was significantly reduced in *P. chabaudi*-infected mice following immunization compared with uninfected, immunized animals. This demonstrates that in infected animals, CD4^+ ^T cells fail to migrate into follicles and therefore fail to interact with antigen-specific B cells, both essential steps in the induction of protective, adaptive immunity. Importantly, these failures in T- and B-cell function are similar to situations in which immune tolerance is induced [56-58], although few studies have directly examined the interaction of T and B cells *in vivo *during the course of infections. It should be noted that, in contrast to the early inflammatory stage of the infection, normal lymphoid architecture was apparent when these defects in migration were observed, with clearly distinguishable, intact B- and T-cell areas (see Figure 9b).

To investigate whether the failure of T-cell migration into follicles was simply due to a potential alteration in lymphoid architecture or chemokine gradients caused by malaria infection, OVA-specific CD4^+ ^T cells were initially activated *in vitro *with bone-marrow-derived DCs and pRBCs and then transferred into uninfected recipient mice. Expansion of T cells stimulated *in vitro *with DCs cultured with infected RBCs was reduced following transfer compared with expansion of OVA-specific T cells activated in the presence of uninfected erythrocytes (Figure 9d), and these cells also failed to migrate into B-cell follicles (data not shown).

To address whether the defect in T-cell migration was due to their activation in the context of parasite infection or to modulation of DC function, highly purified DCs from the spleens of malaria-infected animals or uninfected controls were pulsed with OVA and transferred into naive BALB/c recipient mice along with DO11.10 T cells labeled with the fluorescent dye 5,6-carboxy-succinimidyl-fluorescein ester (CFSE). Following transfer of OVA-pulsed DCs from uninfected mice, T cells divided efficiently, as seen by CFSE dilution (Figure 9e). In mice transferred with OVA-pulsed DCs purified from the spleens of malaria-infected mice, however, OVA-specific CD4^+ ^T cells failed to divide to the same extent (see Figure 9e), suggesting that DCs exposed to malaria parasites are less capable of inducing effective T-cell responses. Furthermore, DCs purified from *in vitro *culture with pRBCs and pulsed with antigen were less able to induce an optimal T-cell response upon transfer to uninfected recipients (data not shown). Thus the defect in T-cell function and migration is primarily due to the modulation of DC function by the malaria parasite, and not simply the result of activation of T cells in the context of infection.

## Discussion

Several previous reports have suggested a generalized suppression of immune responses during infection with *Plasmodium *[2-10,12-21,24-34,59]. One possible mechanism could be impairment of the function of DCs, a cell type that is essential in the generation of the primary immune responses [42]. But the significance of this observation for *in vivo *studies, the implications for downstream immunological function, and what (if any) parasite component mediated this effect remained unclear. Here, we have shown that DCs are modulated by the malaria parasite and are suppressed by infection with *P. chabaudi *through the malarial pigment HZ. Importantly, *Plasmodium *infection also causes a significant defect in the induction of immune responses *in vivo*: expansion and migration of CD4^+ ^T cells is greatly reduced, resulting in a consequent reduction in the interaction between these T cells and B cells and in the help they can provide to the B cells. Despite severely impaired T-cell migration and effector function, early stimulation of antigen-specific CD4^+ ^T cells is not affected by malaria infection, as T cells stimulated *in vitro *and *in vivo *upregulate CD69, suggesting that, despite suppression of DC function, there is sufficient antigen presentation to induce initial T-cell activation.

Although the observed defect in CD4^+ ^T-cell function seems to be directly related to inhibition of DCs, it has also been suggested that optimal T-cell expansion and differentiation requires the interaction of T and B cells [60]. Thus it may be that the failure of DCs to activate properly and subsequently induce T-cell migration into B-cell follicles also results in the reduced expansion of CD4^+ ^T cells observed here. Nevertheless, the failure of optimal CD4^+ ^T-cell expansion and migration during infection clearly results in the abrogation of B-cell expansion and a subsequent absence of antibody production at specific time-points during infection. Thus, protective, systemic immunity against non-parasite antigens, and presumably against parasite antigens, fails to develop effectively.

Although alterations in the architecture of the spleen during infection with *P. chabaudi *have been described [61], these changes were less evident in our model, with intact B220^+ ^B-cell follicles still clearly discernible by day 17 of infection (see Figure 9b). Interestingly, infected mice immunized on day 4 of infection (when lymphoid structure is already disrupted [61]) were able to generate antibody responses, suggesting that despite the breakdown in architecture, immune priming is still normal in these mice.

Our important finding that activated T cells fail to migrate into follicles may in part be due to splenic alterations not visualized in our immunohistochemical staining for B220, or to alteration of chemokine gradients important for T-cell migration in infected mice. To address these issues, we transferred T cells that had been primed *in vitro *in the presence of malaria parasites into uninfected recipient mice with normal lymphoid architecture. In addition, we immunized naive, uninfected mice with OVA-loaded DCs which had been cultured *in vitro *with infected erythrocytes. In all of these situations we could separate the effect of parasites upon DCs or T cells from the described disruption of lymphoid architecture (see Figure 9). In each case, T cells primed in the presence of malaria parasites or T cells primed by DCs exposed to infection failed to differentiate fully following transfer into uninfected recipients. Thus, alteration of splenic architecture alone cannot account for our observations, suggesting that the failure in T-cell differentiation is due to DC modulation.

Another possible explanation for the observed defect in the induction of immunity could be through competition for access to antigen between adoptively transferred OVA-specific T cells and endogenous malarial antigen-specific T cells. Thus, a large dose of blood-borne parasite antigens could impede the induction of other ('bystander') immune responses. But studies examining bystander responses in other parasitic diseases (for example, [62-65]), as well as the potent stimulatory capacity of mycobacteria-containing complete Freunds' adjuvant [66], suggest that such inhibition of immunity does not occur, despite potentially large amounts of competing antigens. In our experiments, naive OVA-specific CD4^+ ^T cells activated *in vitro *and *in vivo *in the presence of infected erythrocytes upregulated CD69 to the same extent as T cells stimulated in uninfected controls, suggesting that sufficient access to antigen was available to initiate T-cell signaling cascades (see Figures 7 and 8). Furthermore, transfer of T cells activated in the presence of infected erythrocytes, or transfer of purified DCs from infected mice into uninfected recipients, transferred the immunosuppressive phenotype, suggesting that the effect cannot be ascribed to out-competition of the OVA-specific T cells by malaria-specific cells (see Figures 8 and 9).

In search of a mechanistic explanation for these observations, we initially focused on analyzing the effect that parasite proteins expressed on the erythrocyte membrane might have on DC function. It is known that the development of the parasites within erythrocytes is coupled with changes in the host cells, including the host-cell plasma membranes [67], and it is well established that parasites express 'neo-proteins' on the host-cell surface, some of which are reported to induce protective immunity [68-70]. Erythrocytes infected with the rodent-specific strain *P. chabaudi *adhere to specific cell types by interacting with molecules such as CD36 [71]; this is known as cytoadherence. Importantly, the interaction of *P. falciparum*-infected RBCs with CD36 has been shown to mediate suppression of DC function [72]. Examination of the *P. chabaudi *genome has not, however, revealed any homologs of the *P. falciparum *protein PfEMP1 [73], which is important in sequestration [72]. Rather, a separate multigene family expressing surface antigens was identified in *P. chabaudi *[73], which may have a role in cytoadherence [74].

Another important difference between *P. chabaudi *(the rodent-specific strain used here) and the human-specific strain *P. falciparum *is the presence of surface complexes, known as knobs, in *P. falciparum*-infected erythrocytes, which strengthen interactions between pRBCs and receptors expressed on other cells [75]. *P. chabaudi*-infected pRBCs have no evident knobs [76]. Thus, although *P. falciparum *and *P. chabaudi *show specific differences in their mechanisms of cytoadherence, pRBC-mediated DC suppression might occur through interactions with membrane molecules, such as CD36. We therefore exposed DCs to erythrocyte ghosts from infected or uninfected erythrocytes, before the LPS challenge. Ghosts isolated from pRBCs did not alter the ability of DCs to respond to LPS treatment *in vitro*, suggesting that parasite antigens expressed on the erythrocyte plasma membranes do not induce the suppression previously described following contact with DCs *in vitro*. In support of this finding is the observation that immunization of mice with pRBC ghosts can induce protection from parasite challenge [77], suggesting that protein structure is maintained on erythrocyte ghosts and that ghosts, unlike intact parasites, are not inherently immunosuppressive.

As fixed pRBCs suppressed the LPS-induced maturation of DCs whereas pRBC membranes did not, HZ seemed to be a good candidate to investigate when looking at the mechanism of parasite-induced modulation of DC function. Previous reports have suggested that HZ impairs the differentiation and functional capacity of human monocytes and murine macrophages through the production of IL-10 and/or the induction of peroxisome proliferator-activated receptor-γ (PPAR-γ) [78-82]. The extrapolation of these results to DCs remains somewhat controversial, however, with other workers suggesting a proinflammatory role for HZ, possibly via the Toll-like receptor-9 [83-86]. Our results suggest that intracellular parasite components, including HZ, do indeed suppress DC maturation and function *in vitro*. The exact mechanism involved in this suppression remains undefined, although it is interesting that phagocytosis of HZ has been found to increase degradation of protein kinase C [87]. Thus, degradation of key intracellular signaling molecules may be one mechanism by which *Plasmodium *parasites suppress DC function. Together, these results suggest that in contrast to *P. falciparum*, intracellular HZ rather than *P. chabaudi*-derived membrane-expressed proteins is responsible for the suppression of APC function.

The results presented here clearly demonstrate that DC function is dynamically modulated *in vitro *and *in vivo *by asexual blood-stage malaria parasites. These findings support previous studies with *P. falciparum*-infected erythrocytes and human monocyte-derived DCs [42] as well as studies *in vitro *and *in vivo *of *Plasmodium yoelii *with murine DCs [88]. Other studies suggest, however, that *P. chabaudi *schizonts activate DCs *in vitro *[40]. Similarly, in previous studies [45,89], DCs isolated from *P. yoelii*-infected mice during peak parasitemia were found to be activated and to efficiently process and present antigen to naive T cells. In the present study, DCs exposed to trophozoite-infected erythrocytes show impaired maturation in response to stimulation, indicating that it is not only different parasites (*P. yoelii *versus *P. chabaudi*) but also different stages (trophozoites versus schizonts) that have different effects on DC function. Interestingly, the observed differences in the ability of pRBCs to stimulate DC maturation may arise through contamination of parasite material with mycoplasmas, which are known to contain potent Toll-like receptor (TLR) ligands that efficiently activate DCs [90-92]).

It has recently been suggested that, during malaria infection *in vivo*, DCs are activated during early infection and then show TLR tolerance later in infection, becoming unresponsive to LPS stimulation [93]. We believe this not to be the case with *P. chabaudi*, however, because we see no evidence of direct maturation of the DCs by the parasite (see Figure 2). In addition, activated DCs show an increased ability to stimulate T-cell proliferation and cytokine production [36], neither of which were observed in the T-cell assays in the current study. Rather, we suggest that the transient increased expression of activation markers on DCs *ex vivo *reflects the high concentrations of pro-inflammatory cytokines caused by the early stage of infection [94]. In support of this, a recent report described that DCs activated through inflammatory cytokines without pathogenic stimulation upregulated markers of activation but were unable to drive CD4^+ ^T-cell differentiation [95].

The ability of DCs to interact with CD40L on T cells *in vivo *has also been used to explain the differences between *in vivo *and *in vitro *studies [45]. Interestingly, in our study we could not rescue DC maturation when pRBC-treated DCs were stimulated with CD40L-transfected fibroblasts, also suggesting that TLR tolerance (the refractory state of DCs to a second stimulation with a TLR ligand) is not involved in the failure of DCs to respond to stimulation. This suggests that the effects that *P. chabaudi*-infected erythrocytes exert on DC function *in vitro *might be more profound than those induced by *P. yoelii *infection. Whether these changes to the CD11c^+ ^population as a whole reflect changes in individual subsets of DCs awaits further investigation. Importantly, we have shown that the immunosuppression seen is due to this inhibition of DC function rather than to suppression of T cells or breakdown of splenic architecture, as transfer of T cells activated in the context of parasites *in vitro *or of DCs from infected mice was sufficient to prevent subsequent T-cell differentiation in uninfected recipients (see Figure 9).

The results presented here demonstrate, for the first time, that suppression of immunity associated with *P. chabaudi *affects multiple populations of cells essential for development of immunity. DC function is impaired during parasite infection, as a result of ingestion of HZ, and although CD4^+ ^T cells specific for a non-parasite antigen become activated following immunization, they fail to expand clonally as efficiently as in uninfected controls. Crucially, these T cells subsequently show a defect in their ability to migrate into B-cell areas and, consequently, fail to provide effective help for B-cell expansion and antibody production. These results demonstrate an overall defect in priming of heterologous immune responses during *Plasmodium *infection and provide an explanation for increased secondary infections and the reduced efficacy of vaccines in areas where malaria is endemic.

## Materials and methods

### Animals and challenge infections

Female BALB/c mice were purchased from Harlan Olac (Bicester, UK). DO11.10 mice, with CD4^+ ^T cells specific for the OVA_323-339 _peptide in the context of the MHC class II molecule I-A^d ^recognized by the KJ1.26 clonotypic antibody [96] were obtained originally from N. Lycke, University of Göteborg, Sweden. MD4 mice containing HEL-specific B cells [51] were backcrossed onto the BALB/c background. All mice were maintained at Biological Services, University of Glasgow, under specific pathogen-free conditions and first used between 6 and 8 weeks of age in accordance with local and UK Home Office regulations.

To initiate a malaria infection, mice were inoculated with 1 × 10^6 ^*P. chabaudi *AS-infected erythrocytes intra-peritoneally. Parasitemia was monitored by thin blood smears stained with Giemsa's stain. Peak parasitemia occurred at 5-6 days post-infection, after which time parasite levels declined and remained at low but usually detectable levels for the remainder of the experiments (see Figure 1a), as previously described [97]. Infected mice were held in a reverse light/dark cycle so that parasites harvested at 08:00 h were at the late trophozoite stage. For studies *in vitro*, blood was collected when parasitemia was 30-40%. Infected blood was recovered into heparin (10 IU/ml) by cardiac puncture and diluted in phosphate-buffered saline (PBS; Invitrogen, Paisley, UK) to the required concentration of pRBCs.

At various times following malaria infection, mice were immunized intravenously with 500 μg OVA (Sigma-Aldrich, Poole, UK), or a conjugate of OVA and HEL (Biozyme, Gwent, UK) [50], along with 50 ng LPS (from *Salmonella equi-abortus*; Sigma-Aldrich).

### Preparation of bone-marrow DCs

DCs were prepared from bone marrow as previously described [98]. Cell suspensions were obtained from femurs and tibias of female BALB/c mice. The bone-marrow cell concentration was adjusted to 5 × 10^5 ^cells/ml and cultured in six-well plates (Corning Costar, New York, USA) in complete RPMI (cRPMI: RPMI 1640 supplemented with L-glutamine (2 mM), penicillin (100 U/ml), streptomycin (100 μg/ml) (all from Invitrogen) and 10% fetal calf serum (FCS; Labtech International, Ringmer, UK) containing 10% of culture supernatant from X63 myeloma cells transfected with mouse granulocyte-macrophage colony stimulating factor (GM-CSF) cDNA. Fresh medium was added to the cell cultures every 3 days. On day 6, DCs were harvested and cultured at the required concentration for each individual experimental procedure, as described below. This technique generated a large number of CD11c^+ ^DCs largely free from granulocyte and monocyte contamination, as previously described [98].

### *In vitro *culture of DCs with fixed infected or uninfected erythrocytes

Blood from *P. chabaudi *AS-infected mice was washed twice in PBS before being resuspended in cRPMI for addition to DCs. For fixation, infected blood was washed three times in PBS and resuspended in 0.5% paraformaldehyde for 30 min at 4°C. Fixed erythrocytes were then washed in PBS, resuspended in 0.06% Gly-Gly (Sigma-Aldrich) for 5 min at 4°C and washed twice more in PBS before being resuspended in cRPMI for addition to DC. After 24 h culture, DCs were stimulated with 1 μg/ml LPS and the expression of cell-surface molecules was analyzed 18 h later by flow cytometry. To confirm complete fixation, we showed that 2 × 10^7 ^fixed, infected erythrocytes could not establish infection when injected intra-peritoneally into a female BALB/c mouse.

### *In vitro *culture of CD40L-transfected fibroblasts with DCs

The cell lines 3T3-CD40L and 3T3-SAMEN [46] were kind gifts from P. Hwu (NCI, Bethesda, USA). Cells were grown in cRPMI in T75 tissue culture flasks (Helena Biosciences, Gateshead, UK) and, when confluent, harvested and distributed in six-well plates at 2.5 × 10^5 ^cells/ml of cRPMI. Bone-marrow-derived DCs were cultured with infected or uninfected erythrocytes at a ratio of 1:100. After 24 h, DCs were harvested, resuspended at 1 × 10^6 ^cells/ml and cultured in a 1:1 ratio with either 3T3-CD40L or 3T3-SAMEN cells for a further 24 h. The level of CD40 expression on DCs was analyzed by flow cytometry and culture supernatants collected for IL-12 cytokine analysis.

### T-cell stimulation *in vitro*

Bone-marrow DCs were centrifuged at 450 × *g*, resuspended at 1 × 10^6 ^cells/ml and 500 μl aliquots were distributed into 24-well tissue culture plates (Corning Costar) with pRBCs or RBCs. After 24 h incubation at 37°C in 5% CO_2_, DCs were antigen-loaded for 6 h with 5 mg/ml OVA (Worthington Biochemical, Freehold, USA). OVA-specific T cells were isolated from the mesenteric and peripheral lymph nodes of DO11.10 transgenic mice [96] on the SCID background and cultured at a 1:1 ratio with DCs. T-cell proliferation was assessed after 48, 72, 96 and 120 h of culture and assessed by incorporation of [^3^H]thymidine (0.5 μCi/well) for the last 24 h of culture. Cells were harvested using a Betaplate 96-well harvester (Wallac Oy, Turku, Finland) and [^3^H]thymidine incorporation measured on a Betaplate liquid scintillation counter (Wallac).

### Cytokine assay

For the detection of IL-12 (p40 and p70) and IL-10, OptEIA™ enzyme-linked immunosorbent assay (ELISA) kits (Becton Dickinson, Oxford, UK) were used according to the manufacturer's instructions. For T-cell cytokines, Mouse Th1/Th2 6-Plex kit (Biosource, Nivelles, Belgium) was used according to the manufacturer's instructions. For analysis of cytokine production *ex vivo*, single-cell suspensions of spleen cells were prepared by rubbing through Nitex mesh (Cadisch & Sons, London, UK) in RPMI 1640 medium. After washing, cells were resuspended at 4 × 10^6 ^cells/ml in cRPMI, either alone or with 1 mg/ml OVA or 5 μg/ml concanavalin A (ConA; Sigma-Aldrich) and supernatants sampled after 48 h. These were stored at -20°C until analysis by standard sandwich ELISA protocol (antibodies used: for IFN-γ capture, R4-6A2; for IFN-γ detection, XMG1.2; for IL-5 capture, TRKF5; for IL-5 detection, TRKF4; Pharmingen, Oxford, UK) and the levels of cytokine in supernatants calculated by comparison with recombinant cytokine standards (R & D Systems, Abingdon, UK).

### Flow cytometry

Aliquots of 1 × 10^6 ^cells in 12 × 75 mm polystyrene tubes (Falcon BD, Oxford, UK) were resuspended in 100 μl FACS buffer (PBS, 2% FCS and 0.05% NaN_3_) containing Fc Block (2.4G2 hybridoma supernatant) as well as the appropriate combinations of the following antibodies: anti-CD4-PerCP (clone RM4-5), anti-CD11c-PE (clone HL3), anti-CD40-FITC (clone 3/23), anti-CD69-PE (clone H1.2F3), anti-CD80-FITC (clone 16-10A1), anti-CD86-FITC (clone GL1), anti-MHC-II (clone 2G9), anti-B220-PE (clone RA3-6B2), PE-hamster IgG isotype control, FITC-rat IgG2a, κ isotype control and FITC-hamster IgG1, λ isotype control (anti-TNP) (all Pharmingen), biotinylated KJ1.26 antibody or biotinylated HEL. Biotinylated antibodies were detected by incubation with fluorochrome-conjugated streptavidin (Pharmingen). After washing, samples were analyzed using a FACSCalibur flow cytometer equipped with a 488 nm argon laser and a 635 nm red diode laser and analyzed using CellQuest software (both BD BioSciences, Oxford, UK).

### Preparation of erythrocyte ghosts from infected and uninfected mouse blood

Ghosts from infected and uninfected erythrocytes were generated as previously described [67]. Briefly, blood was collected into heparin by cardiac puncture and washed three times in PBS. Infected and uninfected erythrocytes were concentrated in PBS supplemented with 113 mM glucose (Sigma-Aldrich) and 3% FCS. Infected erythrocytes were incubated in an equal volume of glycerol buffer (10% glycerol (Sigma-Aldrich) supplemented with 5% FCS in PBS) for 1 h at 4°C. Parasites and ghosts were separated in a continuous Percoll (Amersham Biosciences, Little Chalfont, UK) gradient (ρ: 1.02-1.10 g/cm^3^) in intracellular medium buffer (IM: 20 mM NaCl, 120 mM KCl, 1 mM MgCl_2_, 10 mM glucose, 5 mM Hepes pH 6.7) by centrifugation at 5,000 × *g *for 30 min. Ghosts were then washed in IM buffer and layered on a two-step Percoll gradient (ρ: 1.01+1.02 g/cm^3^) to separate them from ghosts that might still contain parasites. Ghosts from uninfected erythrocytes were obtained by adding a 40-fold volume of phosphate buffer (5 mM NaH_2_PO_4_/Na_2_HPO_4_, 1 mM PMSF, 0.01% azide, pH 8.5). The suspension was centrifuged at 32,000 × *g *for 30 min. Ghosts from infected and uninfected erythrocytes were then washed three times in PBS before being resuspended in cRPMI for addition to DCs at a ratio of 100:1.

### Hemozoin preparation

HZ was isolated from supernatants obtained from cultures of *P. falciparum *gametocytes, kindly provided by Lisa Ranford-Cartwright, (Division of Infection and Immunity, University of Glasgow, UK). Endotoxin-free buffers and solutions were used throughout. Supernatants were centrifuged for 20 min at 450 × *g*. The pellet was washed three times in 2% SLS and resuspended in 6 M guanidine HCl. Following 5-7 washes in PBS, the pellet was resuspended in PBS and sonicated for 90 min using Soniprep 150 (Sanyo Scientific, Bensenville, USA) at an amplitude of 5-8 μm to minimize aggregation and maintain the HZ in suspension. Total heme content was determined as previously described [99] by depolymerizing heme polymer in 1 ml of 20 mM NaOH and 2% SDS, incubating the suspension at room temperature for 2 h and then reading the optical density at 400 nm using a UV-visible Helios spectrophotometer (Thermo Spectronic, Cambridge, UK). DCs were pulsed with 1-20 μM HZ - a concentration range similar to that seen when DCs were cultured at a 1:100 ratio with pRBCs.

### Assessment of antigen-specific antibody responses

Peripheral blood was collected and the plasma was separated by centrifugation at 450 × *g *for 10 min and stored at -20°C until analysis. OVA-specific IgG was measured by standard sandwich ELISA using a peroxidase-conjugated anti-mouse total IgG (Sigma-Aldrich).

### Adoptive transfer of antigen-specific lymphocytes

Lymph nodes and spleens were homogenized and the resulting cell suspensions washed twice by centrifugation at 400 × *g *for 5 min and resuspended in RPMI. The proportions of antigen-specific T cells were evaluated by flow cytometry, and syngeneic recipients received 3 × 10^6 ^antigen-specific cells. In some experiments, cells were labeled with the fluorescent dye CFSE (Molecular Probes, Oregon, USA) immediately before use [100]. The level of CFSE in cells was analyzed by flow cytometry and expressed as the mean proportion of antigen-specific T cells under each CFSE peak.

### Immunohistochemistry

Spleens were frozen in liquid nitrogen in OCT embedding medium (Miles, Elkart, USA) in cryomoulds (Miles) and stored at -70°C. Tissue sections (8 μm) were cut on a cryostat (ThermoShandon, Cheshire, UK) and stored at -20°C. Sections were blocked and stained as previously described [55], using B220-FITC to stain B-cell areas and biotinylated-KJ1.26 to detect OVA-specific DO11.10 cells, and visualized using Streptavidin-Alexa Fluor 647 (Molecular Probes). All photographs were taken at 20× magnification.

To visualize HZ deposition in DCs, cells were photographed using an Axiovert S-100 Zeiss microscope using a 63× oil-immersion lens by normal bright-field imaging. To image HZ in splenic DC, 8 μm sections were cut as described above and stained with biotinylated-CD11c followed by Streptavidin-HRP and finally tyramide-488 (PerkinElmer, Boston, USA). Images were then taken of bright-field and green fluorescence and the images merged by inverting and then false coloring the bright-field image such that deposited HZ appeared red and CD11c appeared green.

### Laser-scanning cytometry

Sections were stained as described above. Sections were then scanned on a laser-scanning cytometer equipped with argon, helium, neon, and ultraviolet lasers (Compucyte, Cambridge, USA) and visualized with the Openlab imaging system (Improvision, Coventry, UK). The localization of transgenic T cells and B-cell follicles were plotted. Using these tissue maps the number of transgenic T cells in defined gates was calculated for three gates in periarteriolar lymphoid sheath (PALS) and three B-cell follicle gates per section. Data are plotted as the mean proportion of transgenic T cells in each gate relative to the number of transgenic T cells in the entire section and are the mean of triplicate readings from three mice per group.

### Isolation of DCs from spleen

Spleens were excised and single-cell suspensions obtained as described above. In some experiments, cells were stimulated with 1 μg/ml LPS for 18 h before analysis by flow cytometry. To obtain purified DCs from spleens of mice, single-cell suspensions were labeled using a CD11c isolation kit (Miltenyi Biotec, Bisley, UK) according to the manufacturer's instructions. DCs were then purified using two MS magnetic columns (Miltenyi Biotec) and found to be 85-95% pure by flow cytometric analysis.

### Statistical analysis

Results are expressed as mean ± standard error or standard deviation as indicated. Significance was determined by one-way ANOVA in conjunction with the Tukey test using Minitab. A *p*-value of less than 0.05 was considered significant.
